# Unexplained Hypercalcemia: A Clue to Adrenal Insufficiency

**DOI:** 10.7759/cureus.42405

**Published:** 2023-07-24

**Authors:** Hezborn M Magacha, Mohammad A Parvez, Venkata Vedantam, Lana Makahleh, Neethu Vedantam

**Affiliations:** 1 Internal Medicine, East Tennessee State University Quillen College of Medicine, Johnson City, USA; 2 Infectious Diseases, East Tennessee State University Quillen College of Medicine, Johnson City, USA

**Keywords:** adrenal crisis, adrenal insufficiency, bone mineral disorder, electrolyte disorders, calcium, hypercalcemia

## Abstract

Hypercalcemia secondary to adrenal insufficiency is a rare condition, but it must be recognized and treated promptly to prevent complications such as kidney damage, bone loss, and cardiac arrhythmias. The co-occurrence of hypercalcemia and adrenal insufficiency can be seen in some rare conditions such as sarcoidosis, however, hypercalcemia as a direct consequence of adrenal insufficiency is well documented in the literature but seldom recognized and often remains underdiagnosed. Symptoms of hypercalcemia in this setting include fatigue, weakness, nausea, vomiting, constipation, abdominal pain, confusion, and dehydration. Treatment typically involves correcting the underlying adrenal insufficiency with hormone replacement therapy, along with measures to lower calcium levels in the blood, such as hydration.

In this article, we report the case of a patient presenting with hypercalcemia secondary to adrenal insufficiency.

## Introduction

Hypercalcemia is a common electrolyte abnormality in both inpatient and outpatient settings. The most common cause of hypercalcemia in patients admitted to the hospital is malignancy, while hyperparathyroidism is the most common cause in the ambulatory setting [1]. The severity of hypercalcemia is directly related to its serum level. Mild hypercalcemia is often asymptomatic, and when it is symptomatic, the manifestations are often vague and can mimic several other diseases [2,3] However, it can sometimes be the only manifestation of an underlying life-threatening disease like malignancy [4]. Hence, it is very important for clinicians to always investigate the etiology of even mild hypercalcemia, as failure to do so can fail to diagnose a potentially fatal underlying pathology. Adrenal insufficiency, though rare, can sometimes manifest as hypercalcemia.

Here we present the case of a 53-year-old female patient who developed acute new-onset hypercalcemia as a manifestation of her secondary adrenal insufficiency.

## Case presentation

A 53 years-old female patient with a past medical history of chronic obstructive pulmonary disease with recurrent exacerbations requiring multiple courses of steroids, recurrent pneumonia, chronic hypoxic respiratory failure on 2 liters of oxygen, active tobacco use, chronic pain syndrome on suboxone, anxiety, and depression presented to the emergency room (ER) with complaints of progressive shortness of breath, cough, and excessive drowsiness of one-week duration. In the ER, the patient was found to be hypotensive with a blood pressure of 70/50 mmHg, saturating at 85% on 6 liters of oxygen. Arterial blood gas (ABG) analysis revealed a pH of 7.25, carbon dioxide (PCO2) of 45 mmHg, and bicarbonate of 20 mmol/L. Chest radiograph revealed multifocal opacities consistent with pneumonia. Septic shock secondary to multifocal pneumonia was diagnosed, and the patient was placed on bilevel positive airway pressure (BiPAP) and transferred to ICU.

**Figure 1:**
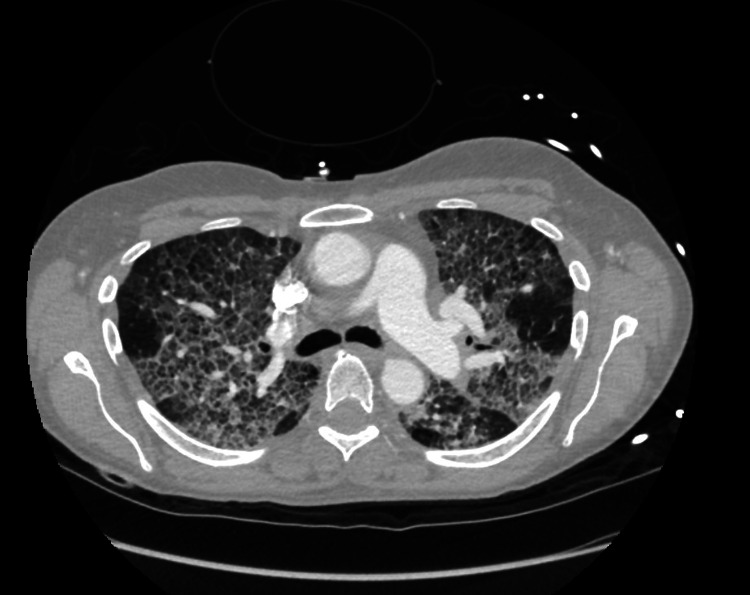
Chest CT scan findings with crazy-paving pattern with interlobular septal thickening

Broad-spectrum antibiotics and intravenous steroids were initiated, and the patient’s blood pressure improved with intravenous fluids. Subsequently, the patient’s mentation improved; however, her oxygen requirement increased to 15 liters on a high-flow cannula. Computed tomography (CT) of the chest revealed a crazy-paving pattern with interlobular septal thickening (Figure 1).

Pulmonology was consulted, and the patient underwent a bronchoscopy, revealing a normal bronchial tree with thin secretions and patchy inflammatory changes. Bronchial washings and pathology did not yield a diagnosis, and periodic acid-Schiff (PAS) staining performed to diagnose pulmonary alveolar proteinosis was unremarkable. Cardiothoracic surgery performed a lung biopsy under video-assisted thoracoscopic surgery (VATS) - results were negative for malignancy. Over the next 7 days, the patient’s symptoms improved significantly, and the patient’s oxygen requirements returned to her baseline of 2 liters. Subsequently, antibiotics and steroids were discontinued.

Two days after discontinuation of steroids the patient started complaining of non-specific diffuse abdominal pain, nausea, and constipation. Her serum chemistry showed sodium trending down from 136 to 131 mEq/L, potassium trending up from 4 to 5.4 mEq/liter, and calcium increased from 8.8 to 11.1 mg/dL. The patient also developed dizziness and orthostatic tachycardia that were unresponsive to intravenous fluids. She denied being diagnosed with prior hypercalcemia by her primary care provider. A review of chemistries 6 months before hospitalization showed normocalcemia. Workup for the acute-onset mild hypercalcemia showed a parathyroid hormone level (PTH) of 30 pg/mL, 25-hydroxy vitamin D level of 45 ng/mL, and phosphorus level of 4.5 mg/dL (Table 1) and normal urine electrolytes. The levels of other pituitary hormones were within normal limits. A CT scan of her chest/abdomen/pelvis did not show any malignancy or osteolytic lesions. The pattern of her chemistries led us to suspect adrenal insufficiency and a workup for the same showed an 8 a.m. cortisol level of 3 mcg/dL and an adrenocorticotropic hormone (ACTH) level of 19 pg/mL. A diagnosis of secondary adrenal insufficiency due to suppression of the hypothalamus-pituitary-adrenal axis was made, and the patient was started on intravenous hydrocortisone with a prompt improvement of her orthostatic symptoms, tachycardia, abdominal pain, and normalization of her sodium, potassium, and calcium levels.

**Table 1:** Showing the results of lab work analysis done from the first day of admission to discharge BUN: blood urea nitrogen; eGFR: estimated glomerular filtration rate; ALT: alanine aminotransferase; AST: aspartate aminotransferase; CO2: carbon dioxide

Laboratory	Reference levels	Patient level				
		Day 1	Day 2	Day 3	Day 4	Discharge Day
Sodium	(136-145) mEq/L	136	135	131	133	135
Potassium	(3.5-5.0) mEq/L	4.3	5.4	5.2	4.5	4.0
Chloride	(95-105) mEq/L	94	94	90	92	99
Glucose	(70-110) mg/dL	88	85	90	135	173
BUN	(7-18) mg/dL	9	9	9	12	11
creatinine	(0.6-1.2) mg/dL	0.55	0.60	0.55	0.46	0.49
Calcium	(8.4-10.2) mg/dL	8.9	10.1	10.6	11.1	9.2
CO2	(33-45) mmHg	33	27	33	28	22
Anion Gap	(3-11) mEq/L	9	14	8	13	14
eGFR	(60-120) ml/min	110	107	110	115	113
Total protein	(6.0-7.8) g/dL	6.9	N/A	7.6	7.4	6.6
Albumin	(3.5-4.5) g/dL	2.8	N/A	2.9	3.0	2.7
ALT	(8-20) U/L	44	N/A	49	52	61
AST	(8-20) U/L	36	N/A	37	38	39
Alkaline Phosphatase	(20-70) U/L	99	N/A	121	116	101
Total Bilirubin	(0.1-1.0) mg/dL	0.3	N/A	0.2	0.1	0.1
Globulin	(2.3-3.5) g/dL	N/A	N/A	4.7	4.4	3.9
Magnesium	(1.5-2) mEq/L	2.0	N/A	2.3	2.1	1.8
Parathyroid Hormone	(10-55) pg/mL	N/A	30			
Vitamin D	(40-60) ng/mL	N/A	45	N/A	N/A	N/A

The patient was eventually discharged with slow tapering of steroids. The patient was educated on symptoms and signs of adrenal insufficiency and was asked to visit the ER if she were to develop any symptoms. Further workup of other possible etiologies of hypercalcemia, including serum protein electrophoresis for paraproteinemia, parathormone-related protein for humoral hypercalcemia of malignancy, and a vitamin 1,25 dihydroxycholecalciferol level, was deferred given the low pretest probability, acute onset, clear temporal correlation with steroid withdrawal, and prompt improvement of symptoms with steroid administration. One week after discharge, a repeat basal metabolic panel (BMP) revealed normocalcemia.

## Discussion

Hypercalcemia is an electrolyte problem that is commonly encountered by physicians in both ambulatory and inpatient settings. Based on severity, it is categorized into mild (serum calcium of 10.5-12 mg/dl), moderate (12.1-14 mg/dl), and severe (>14 mg/dl). The symptoms and signs associated with hypercalcemia depend not only on serum levels but also on the rapidity of onset [4-6]. Mild chronic hypercalcemia is often asymptomatic or can sometimes present with vague symptoms such as fatigue, lethargy, polyuria, dehydration, constipation, and nephrolithiasis.

Acute and severe hypercalcemia is often symptomatic, and if left untreated can progress to obtundation, coma, and life-threatening cardiac arrhythmia. Although many disorders can cause hypercalcemia, hyperparathyroidism and malignancy account for 90% of all cases [1]. Other infrequent causes of hypercalcemia include hypervitaminosis of vitamin D, granulomatous disease, and several commonly used medications such as thiazide diuretics, calcium carbonate, and lithium [7]. Rarely, hypercalcemia can be a consequence of non-parathormone-related endocrinological disorders such as thyrotoxicosis, acromegaly, pheochromocytoma, and adrenal insufficiency [7,8]. Our patient, described in the above clinical scenario, developed acute symptomatic hypercalcemia in the setting of secondary adrenal insufficiency due to the rapid discontinuation of steroids. Our patient likely had underlying adrenal insufficiency due to chronic opioid use, which might have been precipitated by underlying pneumonia and steroid withdrawal.

The prevalence of hypercalcemia in patients with adrenal insufficiency is approximately 6.5% - 8.4% [9]. The exact mechanism of hypercalcemia in patients with adrenal insufficiency is not well understood. Several mechanisms have been postulated as possible explanations including increased bone resorption, increased proximal tubular calcium reabsorption, and increased binding of calcium to serum proteins. Hypovolemia due to adrenal insufficiency may also contribute to hemoconcentration which in turn results in hypercalcemia [8-12]. Adrenal insufficiency can also increase the activity of the renal 1-alpha-hydroxylase enzyme which converts 25(OH)2D to its active form 1,25(OH). Active vitamin D increases the intestinal and renal absorption of calcium, thereby increasing serum calcium levels [9,13]. Hypercalcemia due to adrenal insufficiency has been reported but is not well recognized. Several authors have concluded that the overall prevalence of hypercalcemia in adrenal insufficiency is much higher than reported, for the same reason.

Rushworth and Torpy conducted a study on the trending hospitalizations due to adrenal insufficiency and adrenal crisis between 2000 - 2019. It was noted that admissions due to adrenal insufficiency and adrenal crisis have increased by 62% and 90%, respectively. The study revealed that this increase in incidence is mainly because of a surge in cases of secondary adrenal insufficiency as the overall incidence of primary adrenal insufficiency remained stable [14].Therefore, this increase in the trend of adrenal insufficiency has been mainly attributed to iatrogenic causes following exposure to therapeutic doses of glucocorticoids, including inhaled glucocorticoids, or opioid use, and is often undiagnosed or underdiagnosed [15]. More recently, immunotherapy for treating an increasing number of malignancies is shown to cause hypophysitis, often with selective loss of adrenocorticotropin secretion, or, less commonly, adrenalitis, resulting in adrenal insufficiency in some patients [16,17].

Adrenal crisis is associated with high mortality and the only way to prevent this is by identifying patients at risk and prophylactically treating them with glucocorticoids, however, adrenal crises continue to occur at an approximate rate of six to eight adrenal crises per 100 patient-years and are a cause of increased mortality in adrenal insufficiency [18,19].

One important cause of secondary adrenal insufficiency, which has been well documented in the literature but is seldom recognized by physicians, is adrenal insufficiency due to chronic opioid use. Although the use of prescription opioids has been declining due to increased federal regulations, there has been a parallel increase in the number of prescriptions of buprenorphine to treat patients with opioid use disorder. There are not a lot of adrenal insufficiency cases due to buprenorphine use reported in the literature. However, some case reports suggest a possible association [10,19]. Hence, clinicians need to be more vigilant concerning subclinical suppression of the hypothalamus-pituitary-adrenal axis in all high-risk patients as described above and to commence workup for adrenal insufficiency with morning cortisol and cosyntropin test, even if they have subtle clinical or biochemical abnormalities such as hypercalcemia or hyperkalemia, that would point towards adrenal insufficiency.

## Conclusions

Although hypercalcemia is a relatively common abnormality in all clinical settings, mild hypercalcemia, as noted in our patient, is often not worked up. Irrespective of serum concentration and symptoms, clinicians need to investigate the etiology of hypercalcemia as this can be the only manifestation of an underlying serious illness, such as malignancy. In our case, investigating the cause of our patient’s unexplained hypercalcemia resulted in the diagnosis of adrenal insufficiency. It is also important for primary care physicians and hospitalists to consider adrenal insufficiency as one of the possible causes of their patient’s unexplained hypercalcemia as recognition of this can potentially avert life-threatening adrenal crisis by timely glucocorticoid administration.
